# The Impact of an External Compared to an Internal Focus of Attention on Simple and Complex Motor Skills Acquisition in Novice Female Volleyball Players

**DOI:** 10.3390/children11121560

**Published:** 2024-12-23

**Authors:** Afrodite Lola, Athanasios A. Dalamitros, Aglaia Zafeiroudi, Nur Eradli, Vicente Javier Clemente-Suárez, Alexandra Martín-Rodríguez

**Affiliations:** 1 Laboratory of Motor Behavior and Adapted Physical Activity, Department of Physical Education and Sport Science, Aristotle University of Thessaloniki, Thermi, 57001 Thessaloniki, Greece; 2 Laboratory of Evaluation of Human Biological Performance, Department of Physical Education and Sport Science, Aristotle University of Thessaloniki, 57001 Thessaloniki, Greece; dalammi@phed.auth.gr; 3 Department of Physical Education and Sport Science, University of Thessaly, 42100 Trikala, Greece; azafeiroudi@uth.gr; 4 Management Deparment, Graduate School, Istanbul Technical University, Maslak, 34467 Sariyer, Istanbul, Türkiye; eradli23@itu.edu.tr; 5 Faculty of Sport Sciences, Universidad Europea de Madrid, 28670 Villaviciosa de Odón, Spain; vicentejavier.clemente@universidadeuropea.es (V.J.C.-S.); alexandra.martin@universidadunie.com (A.M.-R.); 6 Grupo de Investigación en Cultura, Educación y Sociedad, Universidad de la Costa, Barranquilla 080002, Colombia

**Keywords:** environmental demands, attention demands, skill complexity, team sports

## Abstract

Objectives: This study aimed to investigate the effects of external (EXT) versus internal (INT) focus of attention on acquiring and transferring simple and complex motor skills in novice female volleyball players. Methods: Fifty-seven participants were assigned to one of the three groups: The external focus (EXT), the internal focus (INT), and the control group (CON). Over a 6-week intervention, participants practiced a simple skill (volleyball passing) and a complex skill (overhead tennis serve). Pre-tests, post-tests, and transfer tests (conducted two weeks later) assessed movement form and accuracy. Results: The results showed a statistically significant interaction among focus methods, skill complexity, and testing periods for movement form and performance outcome. The EXT group showed higher movement form and performance outcome improvements during the simple skill (passing) compared to the INT and CON groups. For the complex skill (serving), the EXT group outperformed the INT and CON groups regarding movement outcome; however, no differences in movement form were observed between the EXT and INT groups. Post hoc Tukey tests indicated that the EXT group scored higher than the INT and CON groups in the post-test and transfer test for the simple skill, with movement form improving from 1.68 ± 0.48 to 2.32 ± 0.58. In the complex skill, the EXT group’s movement outcome improved from 11.32 ± 6.83 to 44.47 ± 16.57. Conclusions: External focus significantly enhances movement form and outcome in simple tasks and performance outcomes in more complex skills among novice athletes.

## 1. Introduction

Properly directing our attention during task execution can enhance performance outcomes, cognitive efficiency, and physiological effectiveness. For instance, individuals often perform better when adopting an external focus of attention (i.e., directing their thoughts toward the effects of their movements on the environment) rather than an internal focus (e.g., concentrating on their bodily movements). Despite these findings, theoretical explanations of these effects have largely been based on hierarchical information-processing frameworks [1].

When novices learn a new skill, they may focus on various elements of the movement pattern or outcome [2]. Instructors are crucial in guiding learners’ attention toward the most relevant information concerning their movement form or outcome [3,4,5]. Numerous studies have supported the positive impact of an external compared to an internal focus of attention on motor skill acquisition [6,7,8,9]. An external focus has been shown to accelerate the learning process, allowing learners to reach a higher skill level more quickly [10], as their movement patterns align with those typically observed in more advanced stages of learning [7].

The “Constrained Action Hypothesis” [11,12] clearly explains the detrimental impact of an internal focus of attention, suggesting that internal focus promotes conscious control over movements, thereby constraining the automatic control processes of the motor system. In the opposite case, an external focus facilitates a more automated process. According to the “Optimal Theory” proposed by Wulf and Lewthwaite, external focus is crucial in linking goals to actions [7]. This theory posits that moving the emphasis away from the body but using an external attentional focus (toward the desired movement outcome or task objective) drives the establishment of effective neural connections critical to effective performance and learning [13,14,15]. The external focus engages the unconscious control process, guiding movements more clearly toward objective-driven actions, strengthening goal-action coupling, and promoting automaticity [11,12]. However, Lawrence et al. suggested that without this, the benefits of external attention may be diminished [16]. While some inconsistencies remain in the literature [17], from a practitioner’s perspective, certain tasks—such as floor gymnastics or dance—may pose challenges in identifying clear external movement effects. To address this issue, Becker et al. recently proposed an innovative solution for these instances through a more comprehensive attentional focus directed toward generalized sensations of movement to suppress conscious control of effectors [18]. This indicates that the focus of attention can be dynamically adapted to optimize performance in various contexts.

In any case, the external focus is not widely adopted in applied sports settings. This disparity may be attributed to two specific factors, namely, the variability of the environment (stable/changed) and the complexity of the sports skills (low/high) or the main task (coordination task/perceptual-motor task, etc.). In sports settings, the primary challenge is often learning “how” to achieve coordinated and skilled control of movements, particularly for complex tasks where the quality of execution is crucial [19]. In this context, Neumman and colleagues observed that performance in activities like rowing improves when athletes alternate their attention between internal and external cues, rather than concentrating exclusively on one type of cue. Furthermore, focusing on external cues diminishes the perception of exertion compared to focusing on internal cues. Therefore, athletes should purposefully shift their attentional focus between internal and external stimuli to maximize their performance [20].

However, other studies [6,8,9] have demonstrated the positive impact of an external focus on learning the movement form and outcome of motor skills across a broad range of tasks. Tapan et al. (2023) reported that the external focus promoted learning of the tennis forehand and backhand in preadolescents [21]. Wulf and Shea (2001), using a dynamic balance task (stabilometer), found that the external focus concluded fewer balance errors compared to an internal focus [22]. Highlighting these results, Wulf (2007) also showed the superiority of an external focus in a balanced skill on surfaces of varying stability [10]. However, contradictory results indicate that the external focus instructions might not be appropriate for tasks that aim to achieve the proper movement technique, as in the case of complex gymnastics routines [16]. Furthermore, Wulf et al. (2002) compared the effectiveness of internal and external focus feedback for learning complex motor skills in novice and expert participants. While the type of feedback did not significantly influence movement quality (form), external focus feedback led to greater accuracy (outcome) in serving, regardless of expertise level, during both the practice and retention phases. These mixed results suggest that more research is required to better understand the effects of an external compared to an internal focus on movement form, particularly in complex motor tasks [21].

Additionally, researchers have emphasized that an external attention focus benefits novice and expert performers across skill levels [5,10]. Singh and Wulf (2020) examined whether external focus can affect the performance of inexperienced and experienced athletes differently [23]. Volleyball players of different skill levels passed a volleyball continuously toward a target. During the proximal focus condition, the participants were instructed to concentrate on the “platform”, while, in the distal focus condition, they concentrated on the target. The high-skilled players performed better in the distal focus condition, demonstrating higher accuracy scores compared to the proximal condition. However, the low-skilled players showed the opposite pattern, with better accuracy in the proximal focus condition. The researchers concluded that the ideal distance for the external focus depends on the expertise level when the skill necessitates a precise movement technique [23]. For low-skilled players, an external focus on movement technique appears more effective. However, focusing on the overall movement effect is more beneficial for high-skilled players as their movement patterns become more automatic. It seems that the impact of an internal and external focus varies based on the level of expertise. Peh et al. (2011) concluded that an external focus had a distinct effect on various phases of learning [24]. Becker and Smith (2013) tested whether factors such as age, gender, and task complexity influence the impact of attentional focus on motor learning [25]. Participants, including children (both boys and girls) and adults (both men and women), were assigned either an internal or external focus and timed while performing a Double Pedalo task, either with handles (representing a simple task) or without handles (representing a complex task). The researchers found that although the external focus improved performance on the more complicated task, no significant differences for the simpler task were noticed. These findings indicate that attentional focus impacts children and adults similarly, yet task complexity probably moderates its effects. Most of the research to date has focused on adult novices or experts. However, for young adults, the primary goal is first to master movement form and then improve the movement outcome of newly learned motor skills. Very few studies have explored the effect of varying attention focuses on young novices, which is one of the key aims of the present study.

Further, different studies have examined the effect of the focus of attention on the performance and learning of complex motor skills, consistently suggesting the superiority of the external focus [26,27]. An et al. (2013) investigated the role of attentional focus instructions on acquiring movement technique and carrying distance in novice golfers, demonstrating that both variables are enhanced in complex skill learning by providing learners with relatively straightforward external focus instructions [28]. Similarly, Abdollahipour et al. (2015) evaluated outcome performance and movement quality in a complex gymnastics task using an internal and an external focus group and a control group. In that case, the external focus achieved superior movement form and outcome (greater jump height) than the rest of the groups [13,17]. However, Tsetseli et al. (2018) found that the external focus positively influenced the internal focus of athletes, which was apparent only for the movement form scores and not the outcome scores [15]. Peh et al. (2011) emphasize the need for future research to explore how different types of instructions can be given toward improving both movement dynamics (form) and movement effects (outcome) during skill acquisition [24]. Moreover, the complexity level may affect the attentional focus factor depending on whether the focus is on movement form or outcome in different ways, suggesting further exploration in this area is warranted. Recent studies, such as those by Bull and colleagues, reported that a distal external focus significantly improved performance and technique compared to an internal focus in a group of skilled cricket batters. In the same way, providing no instructions produced performance benefits similar to those of distal external focus to the same group of participants. In contrast, the internal focus negatively impacted skilled cricket batters in a manner that varies based on the strategic goal of the shot (e.g., protecting the wicket versus attempting to score runs) [29]. Thus, studies with complex tasks in realistic conditions could offer valuable insights into feedback functions that cannot be achieved if the focus is limited to laboratory tasks (e.g., restricted feedback sources) [30]. Furthermore, it is essential to acknowledge that certain methodologies propose that internal and external focus may offer advantages, depending on the specific task requirements and the individual’s abilities.

This study aimed to investigate how different attentional focus strategies impact the performance of young novice female volleyball players, considering skill complexity (low or high) and the specific task conditions in both movement technique and performance outcomes during real-game scenarios. By identifying task characteristics sensitive to attentional focus manipulations, this study seeks to uncover the underlying mechanism(s) contributing to these effects, which are valuable for theoretical understanding and practical application. It is also important, for practical purposes, to enable coaches, physical educators, or therapists to effectively apply the advantages of an external focus for motor skills of different complexity. Although this study focused on female players, any findings might have broader implications for boys’ volleyball and even other sports, as both motor skills involved are fundamentally the same and, in the case of the overhead tennis serve, transferable to other sports. It was hypothesized that an external focus (i.e., focusing on external stimuli, such as the target or the trajectory of the ball) would improve both the movement form (technique) and the performance outcome (accuracy) of simple and complex motor skills, compared to an internal focus of attention (i.e., focusing on body parts). Our hypothesis is based on the expected effect of attentional focus on motor skill learning, with particular attention to the distinctions between simple (passing) and complex (overhead tennis serves) skills. Specifically, the external focus is expected to result in better performance and greater accuracy in these skills due to reduced cognitive load when executing movement. Additionally, improvements are anticipated to be more pronounced in the complex skill, where external focus helps participants manage the demands of coordinating multiple body parts and perceiving spatial and ball movement dynamics.

## 2. Methods

### 2.1. Participants

G*Power software (v. 3.1.9.7.) [31] was applied to calculate a sample size, based on alpha = 0.05, effect size *f* = 0.40 (moderate effect), and 80% statistical power. This procedure concluded that a sample size of 51 participants would meet these criteria. A convenience sampling method was used to recruit participants for this study, consequently involving 57 novice female volleyball players aged 14 to 15 years (mean chronological age: 14.4 years, body mass: 61.3 ± 10.4 kg, body height: 1.69 ± 0.1 m). In this age group, novice female players usually develop technical fundamental volleyball skills [32]. The inclusion criteria required participants not to participate in organized volleyball training and to be free of injuries during the last six months. The participants were randomly and equally assigned to two experimental groups (*n* = 19 for the external—EXT, and *n* = 19 for the internal—INT focus of attention) and the control group (*n* = 19—CON). All of them had recently joined a volleyball sports club and had no prior experience in volleyball. Their participation was voluntary, with the parents’ consent (they signed a consent form before participation) and the coaches’ consent. To ensure the integrity of the results, it was agreed by both researchers and coaches that during this study, participants would not engage in additional practice of the volleyball skills under investigation (serving and passing), so that no external factors would influence the outcomes. The Internal Ethics Committee (IEC) (218/2024) approved this study, which complies with the Declaration of Helsinki.

### 2.2. Experimental Design

The experimental design consisted of both pre-testing and post-testing and an intervention designed to teach participants a simple volleyball task (passing) and a more complex task (overhead tennis serve). The EXT group received explicit instructions to focus on the body parts, while the INT group focused on the target or the ball’s trajectory. After completing the intervention, a test was conducted to evaluate skill acquisition. Additionally, a transfer test was administered two weeks later, without any intervening training, to determine how well participants could apply the learned skills to different task parameters (e.g., varying distance and direction). Both tests and the intervention program were conducted in real-world conditions to ensure ecological validity. Environmental factors such as lighting, noise, temperature, ball condition, court dimensions, and time of day were controlled to ensure consistency and allow participants to focus on the tasks.

### 2.3. Intervention

The intervention lasted for a total of 6 weeks with two 60 min training sessions per week. Each session began with a 10 min warm-up on the volleyball court, followed by 50 min of practicing the following two skills: (a) pass with fingers and (b) overhead tennis serve.

#### Testing Procedure

Each participant executed 23 frontal volleyball passes (the first 3 trials being practiced) and 23 overhead tennis volleyball serves during the testing phase, while the sequence of tasks involved executing the simple motor task before engaging in the complex motor task. The movement evaluation was conducted by two independent raters who used a form with a subjective rating system based on the following three key movement quality criteria for each skill: (a) legs, (b) torso, and (c) frontal gaze for the volleyball pass, and (a) body facing the direction of the serve, (b) movement of the hand (archers’ stance), and (c) movement of the hand hitting the ball for the serve, collecting up to three (3) points. Movement outcome was assessed based on the participants’ accuracy to a target [21,29], with up to three (3) points awarded according to the distance from the target (3 points for 1 m, 2 points for 1–2 m, 1 point for 3 m, and 0 points for over 3 m from the target). The participants did not receive any feedback during the test. Before any testing procedures, the researchers explained the procedure and evaluation method to the participants. The tasks were demonstrated to the participants by a former professional volleyball player. During the transfer measurement, the pass’s direction and distance changed.

The volleyball pass was classified as a relatively simple skill due to its straightforward nature. In this task, learners were required to push the ball to a short target (2.5 m distance) with both hands. In contrast, the volleyball serve was considered a more complex skill, and each participant executed overhead tennis serves to aim at a longer target (9 m distance). The complexity of the volleyball serves stems from several factors. It involves coordinating multiple body parts and joints, necessitates synchronization with a moving object (the ball), and requires spatiotemporal awareness. Furthermore, the serve demands accuracy, perception of the ball’s trajectory, spatial awareness, and shoulder strength. In addition, the diverse targeting adds further difficulty. This skill also places a greater cognitive load on the learner, as it requires more memory retention, decision-making, and processing capacity than the simpler tennis serve, which is less cognitively demanding [21].

### 2.4. Statistical Analysis

All data were tested for normality using the Shapiro–Wilk test, while sphericity assumptions were considered with Mauchly’s Test for each variable. A mixed factorial analysis of variance (ANOVA) (3 methods × 2 complexity × 3 tests) was conducted to evaluate the differences in movement form and movement outcome based on various attentional focus methods and skill complexities across three testing periods. The independent variables included the focus method (with three levels: external focus [EXT], internal focus [INT], and control group [CON]), skill complexity (with two levels: simple skill, which was volleyball passing, and complex skill, which was the overhead tennis serve), and testing period (with three levels: pre-test, post-test, and transfer test). The dependent variables were the movement form, representing the quality of the technique as rated by independent raters, and the movement outcome, indicating the accuracy of the participant’s performance in hitting a target. Repeated measures were applied during the testing period to account for the same participants tested for each of the three measurements. In cases of statistically significant differences, post hoc Tukey tests were conducted, with a significance level set at *p* ≤ 0.05.

## 3. Results

There was a statistically significant interaction among the 3 factors (3 methods × 2 complexity × 3 tests) (F_(4.54)_ = 4.425, *p* < 0.05) as follows: (a) 3 methods × 2 complexity (F_(2.54)_ = 22.804, *p* < 0.05), (b) 3 methods × 3 tests (F_(4.54)_ = 10.066, *p* < 0.05), and (c) 2 complexity × 3 tests (F_(2.54)_ = 14.404, *p* < 0.05). The results are presented in Table 1.

There was a statistically significant main effect of methods (F_(2.54)_ = 21.730, *p* < 0.05) and tests (F_(4.108)_ = 18.638, *p* < 0.05), as well as an interaction among methods and tests (F_(4.108)_ = 18.834, *p* < 0.05) for the movement form of the simple skill. The results from the post hoc tests are presented in Table 2.

There was a statistically significant main effect of methods (F_(2.54)_ = 21.37, *p* < 0.05) and of tests (F_(2.108)_ = 111.01, *p* < 0.05), as well as an interaction among methods and tests (F_(4.108)_ = 21.51, *p* < 0.05) for the movement form of the complex skill. The results from the post hoc tests are presented in Table 3.

There was a statistically significant main effect of methods (F_(2.54)_ = 39.54, *p* < 0.05), of tests (F_(2.108)_ = 99.018, *p* < 0.05), as well as interaction among methods and tests (F_(4.108)_ = 39.34, *p* < 0.05) for the movement outcome of the simple skill. The results from the post hoc tests are presented in Table 4.

There was a statistically significant main effect of methods (F_(2.54)_ = 26.85, *p* < 0.05), of tests (F_(2.108)_ = 81.13, *p* < 0.05), as well as interaction among methods and tests (F_(4.108)_ = 34.86, *p* < 0.05) for the movement outcome of the complex skill. The results from the post hoc tests are presented in Table 5.

## 4. Discussion

This study explored the impact of external versus internal focus instruction on the movement form and outcome of simple and complex volleyball skills among young novice female volleyball players in a real-field condition. According to the results, adopting an external focus of attention can improve both movement technique and performance outcomes in simple skills (volleyball pass) and the movement outcome of the more complex skill (volleyball serve). However, no significant difference was observed between the external and internal focus groups regarding the movement form of the complex skill.

The results presented here are in line with Wulf’s review [6] and Bull’s research [7], which assert that external focus positively affects movement effectiveness, including accuracy, consistency, and balance [6]. Moreover, analyses of movement kinematics and kinetics in various studies suggest that external focus optimizes whole-body coordination patterns [9,29,33,34,35]. Instructions leading to an external focus can enhance learning, contradicting the traditional view that focusing on body movement is essential for learning [29]. Notably, the performance benefits observed were not transient; previous studies by Wulf (2007) and Totsika and Wulf (2003) have shown that external focus leads to superior performance in delayed retention and transfer tests [10,36]. A recent meta-analysis by Chua et al. aligns with previous narrative reviews, demonstrating that an external focus surpasses an internal focus on motor performance and learning, irrespective of age, health status, or skill level [35].

The positive influence of external focus on the movement outcome of complex skills, such as the volleyball serve, aligns with previous research indicating that external focus results in greater accuracy compared to internal focus in various sports, including soccer throw-ins [34], football kicking [37], and long jump [38]. In addition, Wulf and Prinz (2001) explain that the motor system is restricted when internal attentional foci disrupt the body’s natural control processes. This concept is further elaborated in Wulf et al.’s (2001) “Constrained Action Hypothesis”, which posits that an internal focus of attention brings a conscious type of control, causing individuals to constrain their motor system by interfering with automatic control processes [21]. Conversely, an external focus encourages a more automated control mode by operating unconscious, rapid, and reflexive control processes. It has been proposed that by using external focus instructions, the motor system can harness inherent self-organization processes to regulate movements with fewer conscious processes [39]. Other studies have shown that external focus instructions are associated with increased automaticity, characterized by reduced demands on attentional capacity, high-frequency movement adjustments, and shorter pre-movement times, all contributing to more efficient motor planning [40].

In this study, no significant difference was found between the external and internal focus groups regarding movement form for the complex skill of the volleyball serve, which contrasts with previous research manipulating attention to external and internal features [6]. This disparity between the current results and prior studies is probably due to the different complexity levels of this task. It was found that the movement form scores of both the external and internal attention focus groups showed a small improvement from pre-to-post and transfer tests. However, participants in the external focus group were asked to focus on the ball’s trajectory or the target while executing a complex skill. It is possible that asking participants to focus on trajectory or outcome while performing an attention-demanding complex skill exceeded their attentional capacity and restricted their improvement. Additionally, the lack of declarative knowledge probably decreased the movement form scores of the external focus group. Although the internal focus group participants were instructed to focus on a single point (of their body part), they still had to execute a complex skill that demanded much attention. The internal focus of attention participants received declarative knowledge of how to perform the movement form of the complex skill. However, their efforts to implement the instructions disrupted the automatic control processes of the motor system, decreased the goal-action coupling, and decreased the improvement in movement form. These findings are consistent with previous studies that explicitly assessed movement form [16], and it appears that when the tasks are assessed on the accurate production of a particular movement pattern/technique or form, the benefits of adopting an external focus cannot be generalized. Peh et al. (2011) assert that the internal focus of attention may also be useful to learners if the performance context emphasizes movement form instead of performance outcomes [24]. Also, Aiken and Becker highlighted that the adverse effects of an internal focus are observed solely during skill execution, not during movement preparation. Learners can gain advantages from an internal and external focus, provided that the cues are delivered during the appropriate phase of skill execution [41].

The external focus is not frequently adopted in applied sports settings when the primary goal is to improve movement form. Similarly, Lawrence et al. (2011) propose that the external focus of instructions may not be appropriate for tasks in which the main objective is to produce the correct movement form [16]. Supporting this, studies by Maddox et al. (1999) and Wulf, McNevin, and Shea (2001) found no difference in movement form when ratings to evaluate movement quality under external versus internal attentional focus conditions [11]. As Wulf et al. (2002) remark, “In the acquisition of complex motor skills with many degrees of freedom, such as the ones used here, where the performer’s major goal is the learning of the correct technique, it might be almost impossible to come up with statements that do not refer at all to the performer’s movements” [30]. Lawrence et al. (2011) similarly argue that “the benefits of adopting an external focus do not generalize to tasks that are assessed on the accurate production of a particular movement pattern/technique or form” [16]. Perhaps focusing on the movement technique, while not concentrating on the movements per se, might be more beneficial than focusing on the outcome directly. This may be especially true for novices attempting to acquire a relatively complex skill like the one used here. Peh et al. (2011) also propose that “an internal focus of attention may be more useful to the learner if the performance context emphasizes movement form instead of performance outcomes”. However, further research is needed on tasks emphasizing specific movement forms’ production [24]. Lawrence et al. (2011) similarly highlight that external focus instructions might not be appropriate for tasks that aim to produce the correct movement form, such as gymnastics routines [16]. However, an ecological perspective on the focus of attention, derived from Davies and Davies [42], Newell’s [43] constraints model, and Wulf and Lewthwaite’s OPTIMAL model [7,44], suggests that the focus of attention should be seen concerning the interactions between the individual and their environment. This method posits that internal and external focuses may be advantageous based on task requirements and individual competencies. Rather than considering the focus of attention as a discrete variable, it underscores the significance of the individual’s perception and reaction to pertinent information in their surroundings. This adaptive approach indicates that the concentration of attention can be flexibly adjusted to enhance performance in various contexts.

In this study, all tests were conducted in a real sports environment, which is beneficial for the practical application of the findings. However, an obvious drawback is that the long-term assessment of skill retention was not included in the experimental design. Evaluating retention over time is critical to understanding whether the acquired skills are sustained or diminished after the intervention. Future studies should incorporate long-term retention assessments to better gauge the lasting impact of different focus of attention strategies on skill acquisition and performance.

The novelty of this study lies in its targeted focus on novice female volleyball players aged 14 to 15 years, a demographic often underrepresented in sports research, particularly in motor skill learning and attentional focus strategies. By concentrating on this specific age group, this study provides valuable insights into the development of motor skills in young female athletes, addressing their unique learning needs and experiences in volleyball. Furthermore, this study explores the effects of external versus internal attention focus across different skill complexity levels by examining simple (volleyball pass) and complex (overhead tennis serves) skills. This dual approach enriches the existing literature by demonstrating how attentional focus can be customized for different skill types, an aspect often overlooked in previous studies.

Conducting the research in a real-world field setting, rather than in a controlled laboratory environment, adds practical relevance to the findings, making them directly applicable to coaches and trainers. Many studies in the field have been limited to controlled conditions that may not fully capture the complexities and pressures athletes face in actual sports environments. This study provides actionable insights that may enhance coaching practices and athlete training programs by assessing attentional focus in realistic training contexts. Additionally, the findings contribute to theoretical frameworks such as the Constrained Action Hypothesis and the Optimal Theory of Motor Learning. By demonstrating that an external focus can improve both movement form and outcome, particularly for novice athletes, the research advances our understanding of how attentional focus strategies influence motor skill acquisition in team sports.

Moreover, the implications for skill acquisition are significant, suggesting that adopting an external focus benefits learning simple skills and positively influences the outcomes of more complex skills. This insight highlights the need for coaches to consider skill complexity when designing instructional strategies, potentially leading to more effective training regimens that enhance athletic performance. Finally, this study lays the foundation for future research by identifying gaps in the literature regarding the attentional focus on young female athletes. It further explores how competitive experience, environmental factors, and psychological aspects (e.g., anxiety levels) interact with attentional focus and skill acquisition. Overall, the novel contributions of this research not only advance academic understanding but also have practical implications for coaching practices, ultimately fostering the development of young female athletes in volleyball and beyond. However, some limitations associated with this study should be acknowledged, for example, that only female participants of a specific age group were investigated. In addition, fatigue is often more pronounced in novice players due to their lack of developed technique. Finally, the inter-rater reliability regarding the movement form was not determined. Future studies should, additionally, investigate the long-term retention of basic or more complex volleyball skills, apart from those examined here, along with strategies that utilize different attention focuses. Finally, it would be useful to analyze gender- and age-related differences that possibly arise due to biomechanical differences and physical attributes, along with changes in kinematics due to fatigue. Addressing these limitations will help extend this study’s applicability and methodological rigor.

### Practical Applications

- Tailored Coaching Strategies: Coaches can use the insights from this study to design training programs that specifically cater to the unique learning needs of novice female volleyball players. Implementing small-sided games may encourage focus on positioning and game dynamics, fostering reaction to external stimuli.
- Skill Development Programs: Sports organizations and schools can develop skill development programs that incorporate the findings on attentional focus. These programs can be structured to gradually introduce more complex skills as athletes improve, ensuring that the focus of attention is appropriately adjusted to enhance learning.
- Real-World Training Adaptations: Trainers and coaches can apply the practical insights from this study’s real-world field setting to make training sessions more effective. Simulating game conditions and pressures can help athletes transfer their skills from practice to competition.
- Individualized Attention: Coaches can individualize their attention strategies based on the athlete’s skill level and the complexity of the skill being learned, along with setting specific goals (e.g., achieve a percentage improvement in successful serves within a given timeframe).
- Cross-Sport Applications: While this study focuses on volleyball, the findings can be applied to other team sports. Coaches in sports like basketball, soccer, or field hockey can adapt the strategies to benefit their athletes.
- Athlete Confidence and Anxiety Management: Understanding the impact of attentional focus on performance can help athletes manage their anxiety levels and build confidence.

## 5. Conclusions

In conclusion, it appears that the adoption of an external focus of attention (directing attention to the target or ball trajectory) has a positive effect on both movement form and movement outcome for simple sports skills as well as on the movement outcome for more complex skills in novice female volleyball players. This is the case in both the movement form and the movement outcome of the sports skill. However, when learning a more complex novel sports skill, adopting either an internal (directing attention to body parts) or an external focus of attention instruction method improves movement form. This is because both methods direct attention to different parts of the body. As a result of the ambiguity of the findings regarding the positive effects of external focus on the movement form of complex skills, additional research is required to determine the positive effects of external focus on movement form for sports skills. Additionally, due to the varying complexity of skills within the same sport, the constraints of different tasks, the diverse demands of sports environments, the various evaluation criteria (movement form or outcome), and the practice goals in the other learning stages, more research is required to reevaluate whether or not the external focus of attention instructions is beneficial across different sports contexts.

## Figures and Tables

**Table 1:** Factorial ANOVA (between-subjects’ effects) for methods, tests, and complexity regarding the movement form and outcome.

Source	Dependent Variable	Sum of Squares	df	Mean Square	F	Significance
Method	Form	11.57	2	5.78	100.21	0.000
	Outcome	4.68	2	2.34	86.72	0.000
Complexity	Form	0.09	1	0.09	1.56	0.213
	Outcome	296.05	1	296.05	10,971.44	0.000
Test	Form	7.67	2	3.83	66.48	0.000
	Outcome	2.21	2	1.10	41.07	0.000
Method × complexity	Form	0.05	2	0.029	0.50	0.603
	Outcome	2.50	2	1.25	46.44	0.000
Method × test	Form	3.36	4	0.84	14.57	0.000
	Outcome	1.54	4	0.38	14.27	0.000
Complexity × test	Form	0.13	2	0.06	1.16	0.314
	Outcome	1.38	2	0.69	25.59	0.000
Method × complexity × test	Form	0.04	4	0.01	0.19	0.939
	Outcome	0.88	4	0.22	8.15	0.000

**Table 2:** Analyses of the main effects of post hoc Tukey tests among the methods and tests in the simple skill (passing) regarding the movement form.

	External (ex)		Internal (in)		Control (co)		Post-Hoc
	*M*	*SD*	*M*	*SD*	*M*	*SD*	
Pre-test (Pre)	1.68	0.48	1.74	0.56	1.79	0.63	ex = in = co
Post-test (Post)	2.32	0.58	1.95	0.23	1.68	0.58	ex > in > co
Transfer test (T)	2.21	0.44	1.89	0.32	1.68	0.67	ex > in > co
Post hoc	Pre < Post, Post = Τ		Pre < Post, Post = Τ		Pre = Post, Post = Τ		

**Table 3:** Analyses of the main effects of post hoc Tukey tests among the methods and tests in the complex skill (serving) regarding the movement form.

	External (ex)		Internal (in)		Control (co)		Post-Hoc
	*M*	*SD*	*M*	*SD*	*M*	*SD*	
Pre-test (Pre)	1.79	0.42	1.68	0.58	1.63	0.50	ex = in = co
Post-test (Post)	2.37	0.50	2.42	0.61	1.68	0.67	ex = in > co
Transfer test (T)	2.26	0.56	2.21	0.42	1.74	0.56	ex = in > co
Post hoc	Pre < Post, Post = Τ		Pre < Post, Post = Τ		Pre = Post, Post = Τ		

**Table 4:** Analyses of the main effects of post hoc Tukey tests among the methods and tests in the simple skill (passing) regarding movement outcome.

	External (ex)		Internal (in)		Control (co)		Post-Hoc
	*M*	*SD*	*M*	*SD*	*M*	*SD*	
Pre-test (Pre)	2.21	0.54	2.16	0.50	2.26	0.65	ex = in = co
Post-test (Post)	2.89	0.32	2.42	0.51	2.21	0.63	ex > in > co
Transfer test (T)	2.84	0.50	2.32	0.67	2.32	0.58	ex > in > co
Post hoc	Pre < Post, Post = Τ		Pre < Post, Post = Τ		Pre = Post, Post = Τ		

**Table 5:** Analyses of the main effects of post hoc Tukey tests among the methods and tests in the complex skill (serving) regarding the movement outcome.

	External (ex)		Internal (in)		Control (co)		Post-Hoc
	*M*	*SD*	*M*	*SD*	*M*	*SD*	
Pre-test (Pre)	11.32	6.83	11.37	7.78	10.26	8.57	ex = in = co
Post-test (Post)	44.47	16.57	22.16	11.21	12.32	6.74	ex > in > co
Transfer test (T)	42.89	16.77	19.21	11.57	11.53	7.63	ex > in > co
Post hoc	Pre < Post, Post = Τ		Pre < Post, Post = Τ		Pre = Post, Post = Τ		

## Data Availability

Data are contained within the article.

## References

[1] Gottwald V., Davies M., Owen R. (2023). Every Story Has Two Sides: Evaluating Information Processing and Ecological Dynamics Perspectives of Focus of Attention in Skill Acquisition. Front. Sports Act. Living.

[2] Wulf G., McNevin N.H., Fuchs T., Ritter F., Toole T. (2000). Attentional Focus in Complex Skill Learning. Res. Q. Exerc. Sport.

[3] Beilock S.L., Bertenthal B.I., Mccoy A.M., Carr T.H. (2004). Haste Does Not Always Make Waste: Expertise, Direction of Attention, and Speed versus Accuracy in Performing Sensorimotor Skills. Psychon. Bull. Rev..

[4] Ford P., Hodges N.J., Williams A.M. (2005). Online Attentional-Focus Manipulations in a Soccer-Dribbling Task: Implications for the Proceduralization of Motor Skills. J. Mot. Behav..

[5] Wulf G., Höß M., Prinz W. (1998). Instructions for Motor Learning: Differential Effects of Internal versus External Focus of Attention. J. Mot. Behav..

[6] Wulf G. (2013). Attentional Focus and Motor Learning: A Review of 15 Years. Int. Rev. Sport Exerc. Psychol..

[7] Wulf G., Lewthwaite R. (2016). Optimizing Performance through Intrinsic Motivation and Attention for Learning: The OPTIMAL Theory of Motor Learning. Psychon. Bull. Rev..

[8] Marchant D.C., Greig M., Bullough J., Hitchen D. (2011). Instructions to Adopt an External Focus Enhance Muscular Endurance. Res. Q. Exerc. Sport.

[9] Lohse K.R., Sherwood D.E., Healy A.F. (2010). How Changing the Focus of Attention Affects Performance, Kinematics, and Electromyography in Dart Throwing. Hum. Mov. Sci..

[10] Wulf G. (2007). Attention and Motor Skill Learning.

[11] Wulf G., McNevin N., Shea C.H. (2001). The Automaticity of Complex Motor Skill Learning as a Function of Attentional Focus. Q. J. Exp. Psychol. A.

[12] Wulf G., Shea C., Park J.H. (2001). Attention and Motor Performance: Preferences for and Advantages of an External Focus. Res. Q. Exerc. Sport.

[13] Abdollahipour R., Palomo Nieto M., Psotta R., Wulf G. (2017). External Focus of Attention and Autonomy Support Have Additive Benefits for Motor Performance in Children. Psychol. Sport Exerc..

[14] Tsetseli M., Zetou E., Vernadakis N., Michalopoulou M. (2016). The Effect of Internal and External Focus of Attention on Game Performance in Tennis. Acta Gymnica.

[15] Tsetseli M., Zetou E., Vernadakis N., Mountaki F. (2018). The Attentional Focus Impact on Tennis Skills’ Technique in 10 and under Years Old Players: Implications for Real Game Situations. J. Hum. Sport Exerc..

[16] Lawrence G.R., Gottwald V.M., Hardy J., Khan M.A. (2011). Internal and External Focus of Attention in a Novice Form Sport. Res. Q. Exerc. Sport.

[17] Abdollahipour R., Wulf G., Psotta R., Palomo Nieto M. (2015). Performance of Gymnastics Skill Benefits from an External Focus of Attention. J. Sports Sci..

[18] Becker K., Georges A., Aiken C. (2018). Considering a Holistic Focus of Attention as an Alternative to an External Focus. J. Mot. Learn. Dev..

[19] Shmuelof L., Krakauer J.W. (2011). Are We Ready for a Natural History of Motor Learning?. Neuron.

[20] Neumann D.L., Olive A., Moffitt R.L., Piatkowski T. (2022). Switching Attentional Focus across Internal and External Cues Improves Performance in a Rowing Task in Novices. Psychol. Sport Exerc..

[21] Tapan T., Şahan A., Erman K.A. (2023). The effect of internal and external focus of attention on tennis skill acquisition in children. Front. Psychol..

[22] Wulf G., Shea C.H. (2002). Principles derived from the study of simple skills do not generalize to complex skill learning. Psychon. Bull. Rev..

[23] Singh H., Wulf G. (2022). Mind over Body: Creating an External Focus for Sport Skills. Eur. J. Sport Sci..

[24] Peh S.Y.-C., Chow J.Y., Davids K. (2011). Focus of Attention and Its Impact on Movement Behaviour. J. Sci. Med. Sport.

[25] Becker K., Smith P.J.K. (2013). Age, Task Complexity, and Sex as Potential Moderators of Attentional Focus Effects. Percept. Mot. Skills.

[26] Singer R.N., Lidor R., Cauraugh J.H. (1994). Focus of Attention during Motor Skill Performance. J. Sports Sci..

[27] Wulf G., Weigelt C. (1997). Instructions about Physical Principles in Learning a Complex Motor Skill: To Tell or Not to Tell. Res. Q. Exerc. Sport.

[28] An J., Wulf G., Kim S. (2013). Increased Carry Distance and X-Factor Stretch in Golf Through an External Focus of Attention. J. Mot. Learn. Dev..

[29] Bull H.G., Atack A.C., North J.S., Murphy C.P. (2023). The Effect of Attentional Focus Instructions on Performance and Technique in a Complex Open Skill. Eur. J. Sport Sci..

[30] Wulf G., McConnel N., Gärtner M., Schwarz A. (2002). Enhancing the Learning of Sport Skills through External-Focus Feedback. J. Mot. Behav..

[31] Faul F., Erdfelder E., Lang A.-G., Buchner A.G. (2007). Power 3: A flexible statistical power analysis program for the social, behavioral, and biomedical sciences. Behav. Res. Methods.

[32] Ntozis C., Barzoukal K., Skouras A.Z., Cherouveim E., Papitsi F., Apostolidis N., Tsolakis C. (2024). Relative Age Effect for Different Playing Positions in Adolescent Female Volleyball Players. Int. J. Exerc. Sci..

[33] Parr R., Button C. (2009). End-Point Focus of Attention: Learning the “Catch” in Rowing. Int. J. Sport Psychol..

[34] Wulf G., Chiviacowsky S., Schiller E., Avila L.T.G. (2010). Frequent External-Focus Feedback Enhances Motor Learning. Front. Psychol..

[35] Chua L.-K., Jimenez-Diaz J., Lewthwaite R., Kim T., Wulf G. (2021). Superiority of External Attentional Focus for Motor Performance and Learning: Systematic Reviews and Meta-Analyses. Psychol. Bull..

[36] Totsika V., Wulf G. (2003). The Influence of External and Internal Foci of Attention on Transfer to Novel Situations and Skills. Res. Q. Exerc. Sport.

[37] Zachry T.L. (2005). Effects of Attentional Focus on Kinematics and Muscle Activation Patterns as a Function of Expertise. Master’s Thesis.

[38] Jong C.D., DeBeliso M. (2020). The effects of internal and external focus attention on the standing long jump among high school female athletes. Eur. J. Phys. Educ. Sport Sci..

[39] Davids K., Button C., Bennett S. (2008). Dynamics of Skill Acquisition: A Constraints-Led Approach.

[40] Carpenter S.K., Lohse K.R., Healy A.F., Bourne L.E., Clegg B.A. (2013). External Focus of Attention Improves Performance in a Speeded Aiming Task. J. Appl. Res. Mem. Cogn..

[41] Aiken C.A., Becker K.A. (2023). Utilising an Internal Focus of Attention during Preparation and an External Focus during Execution May Facilitate Motor Learning. Eur. J. Sport Sci..

[42] Davies M., Davies R. Developing Adaptive Expertise in Exploration Decision-Making. Proceedings of the 15th Biennial Meeting of the Society for Geology Applied to Mineral Deposits.

[43] Newell K.M., Goodman D., Wilberg R.B., Franks I.M. (1985). Coordination, Control and Skill. Differing Perspectives in Motor Learning, Memory and Control.

[44] Lewthwaite R. (2021). Translating Thoughts into Action: Optimizing Motor Performance and Learning Through Brief Motivational and Attentional Influences. Curr. Dir. Psychol. Sci..

